# Lead: Washington’s Water Woes

**DOI:** 10.1289/ehp.112-a735a

**Published:** 2004-09

**Authors:** Tina Adler

For at least two years the concentration of lead in Washington, D.C., drinking water has dramatically exceeded the action level at which the Safe Drinking Water Act requires water systems to address the problem. By this summer, additional steps had been taken to address water quality through treatment, but these steps will take months to become fully effective. Indeed, the controversy surrounding the problem resembles the plot of a political potboiler, and blood tests and water filters are still hot topics among Washingtonians.

Of approximately 130,000 residences served by the District of Columbia Water and Sewer Authority (DCWASA), an estimated 18% have lead service pipes. Lead is in some older solder and plumbing fixtures as well. Paint and dust remain the main sources of lead exposure in the United States, but on average 10–20% of U.S. environmental lead exposure comes from drinking water, according to the EPA. (Experts largely agree, however, that the Safe Drinking Water Act amendments have greatly reduced exposure from the lead service pipes that still serve many households in older communities throughout the country.) Lead exposure impairs intellectual and physical development in fetuses and young children. In adults, it appears to increase the risk for hypertension and kidney disease.

Under the Lead and Copper Rule of the U.S. Environmental Protection Agency (EPA), water systems are required to develop a plan to lower lead levels if 10% of residences tested exceed 15 parts per billion (ppb). According to Alexandra Teitz, minority counsel for the House Committee on Government Reform, 73% of one set of water samples from Washing-ton homes exceeded the action level, with numerous samples exceeding 100 ppb and some exceeding 300 ppb. Moreover, before 2002, DCWASA was required to test only 50 residences each year.

Washington’s recent water quality troubles may have begun as early as November 2000. That’s when health officials, with the EPA’s approval, stopped using chlorine disinfection because of its by-products. The city switched to a chlorine–ammonia compound called chloramine to disinfect the water, while using pH adjustments to control corrosion. Unbeknownst to scientists and water utilities at the time, says Johnnie Hemphill, interim director for public affairs at DCWASA, pH adjustments are not as effective without chlorine. The absence of chlorine was not implicated until 2004—water system officials used chlorine in April and May of that year, and lead levels temporarily dropped, says Hemphill.

Consumers were first informed of the elevated lead levels in October 2002 via water bill inserts and a mailed brochure—means that some critics say tended to downplay the situation. As Hemphill explains it, the EPA then demanded that DCWASA explain whether it had failed to adequately monitor for lead or to adequately alert the public and the EPA about the elevated levels.

At the same time, members of Congress charged the EPA with failing to adequately protect the country’s drinking water. “The District and its residents were unknowingly forced to serve as a ‘canary in the coal mine’ for lead in drinking water,” asserted Representative Henry Waxman (D–California) in a statement presented at a congressional hearing in May 2004. “We have now been clearly warned about the flaws in our national program on lead in drinking water.”

In June, officials in Washington began adding phosphoric acid, a food additive, to a small portion of the system to protect the pipes. In July, DCWASA accelerated its timetable for replacing its lead service lines, promising to complete the job by 2010 (under EPA regulations, water systems need replace only a small percentage of public service lines per year and may approve lines using lead testing in lieu of actual pipe replacement). The city is offering loans to those residents who want to replace the part of the line on their property, which is the homeowner’s responsibility.

Blood tests, which the city has offered for free to residents, are indicating that the number of Washingtonians with high blood lead levels has not increased, Hemphill says. But this good news is overshadowed by studies showing that even at blood levels below the current cutoff of 10 micrograms per deciliter (μg/dL), lead can lower children’s IQ and cause behavior problems, says Lynn Goldman, an environmental health scientist at The Johns Hopkins University. A task force from the Centers for Disease Control and Prevention is considering recommending that the cutoff be lowered to 5 μg/dL, although Goldman notes that many experts think there is no threshold for the toxic effects of lead.

